# Deciphering The Emerging Role of Programmed Cell Death in Diabetic Wound Healing

**DOI:** 10.7150/ijbs.88461

**Published:** 2023-09-18

**Authors:** Jingyu Song, Keyu Zhu, Haiping Wang, Min Wu, Yiping Wu, Qi Zhang

**Affiliations:** Department of Plastic and Cosmetic Surgery, Tongji Hospital, Tongji Medical College, Huazhong University of Science and Technology, Wuhan, 430030, China.

**Keywords:** Diabetic Wound Healing, Programmed Cell Death, Apoptosis, Autophagy.

## Abstract

Diabetic wounds are characterized by delayed and incomplete healing. As one of the most common complications of diabetes, diabetic wounds can be fatal in some cases. Programmed cell death (PCD) is an active and ordered cell death mode determined by genes, including apoptosis, autophagy, pyroptosis, necroptosis, ferroptosis, and cuproptosis. It is currently believed that PCD plays a crucial role in diabetic wound healing. Diabetic hyperglycemic environments can lead to abnormal PCD in various cells during healing processes, thereby affecting the activity and function of cells and interfering with diabetic wound healing. Therefore, this review focuses on the new roles and mechanisms of PCD in diabetic wound healing. Moreover, the challenges and perspectives related to PCD in diabetic wound healing are presented, which will bring new insights to improve diabetic wound healing.

## 1. Introduction

Wound healing is a dynamic process that plays an important role in protecting the human body from various harmful external factors, mainly including hemostasis, inflammation, proliferation, and remodeling processes 1. These phases overlap in space and time and involve the coordination of various cells, including neutrophils, macrophages, keratinocytes (KCs), endothelial cells (ECs), endothelial progenitor cells (EPCs), fibroblasts, and others 2,3. Wound healing is a very complex biological process disturbed by multiple physiological and pathological factors. Currently, diabetes is recognized as one of the leading causes of poor wound healing and chronic non-healing ulcers 4. With the number of people with diabetes rapidly increasing worldwide, reaching 642 million by 2040, diabetic wounds will constitute a major issue threatening the life quality of people with diabetes 5. The current lack of very effective tools in the treatment of diabetic wounds is intimately related to the complex pathogenesis of diabetic wounds. Therefore, a thorough understanding of the changes in various cells involved in diabetic wound healing will help to find novel targets for ameliorative diabetic wound healing.

Cell death is an irreversible process of cells caused by exogenous or endogenous damage factors 6. At first, cell death was classified into two types, necrosis, and apoptosis. Necrosis represents the passive death of cells, while apoptosis refers to the active death of cells under the regulation of related genes 7. Later, other types of active and ordered cell death, including autophagy, pyroptosis, and necroptosis, are confirmed and are collectively referred to as programmed cell death (PCD) 8,9. In general, these PCD modalities can be divided into two categories based on the presence or absence of cell lysis and extravasation of cellular contents in terms of morphological changes. One of them is represented by apoptosis and autophagy, which do not cause cell rupture and extravasation of cell contents. Specifically, apoptotic cells eventually form apoptotic bodies and are phagocytosed by other cells, while autophagy transports intracellular substances to lysosomes for degradation. This type of “silent” PCD does not cause an inflammatory response around the cell. In contrast, the other category is represented by pyroptosis and necroptosis, which are both typically characterized by eventual cell lysis accompanied by releasing a large number of pro-inflammatory factors. It is worth noting that pyroptosis is caused by the activation of inflammasome sensors, such as the Nod-like receptor (NLR) family, Absent in Melanoma 2 (AIM2) and the pyrin receptor, while necroptosis is usually secondary to a blocked apoptotic pathway 10. In recent years, ferroptosis and cuproptosis have attracted great attention as newly discovered types of PCD. Ferroptosis refers to the occurrence of lipid peroxidation in cells under the action of divalent iron, along with a decrease in the expression of antioxidants such as Glutathione (GSH) and Glutathione Peroxidase 4 (GPX4), thus inducing cell death. The main mechanism of cuproptosis is the direct binding of copper ions to the fatty acylated components of the Tricarboxylic Acid (TCA) cycle, leading to disruption of the TCA cycle, which in turn triggers proteotoxic stress and induces cell death 11,12.

As an important biological process *in vivo*, PCD plays an important role in a variety of physiological and pathological conditions. Under normal conditions, PCD removes unwanted or abnormal cells to maintain homeostasis, while in pathological conditions, abnormal PCD can cause excessive cell death or disrupt coordination and balance between cells, thus promoting the development of disease 13. In diabetic patients, the abnormal high-glucose environment in the body is one of the most important pathological features 14. Therefore, when wounds occur in the skin of diabetic patients, the various cells involved in wound healing are affected by the high-glucose environment, resulting in abnormal cell function and PCD, which are key causes of poor wound healing in diabetes 15,16. Currently, there is increasing evidence that PCD plays an important role in diabetic wound healing. In this review, we will focus on the recent research progress on the roles and mechanisms of PCD in diabetic wound healing, and shed light on the relationship between various types of PCD and diabetic wound healing, thus greatly aiding in the development of novel and effective targets and drugs.

## 2. Roles of apoptosis in diabetic wound healing

Apoptosis is the most familiar form of PCD and is characterized by a series of nuclear morphological changes, including nuclear pyknosis, nuclear membrane breakdown, and DNA fragmentation. Apoptotic cells are eventually packaged into several apoptotic bodies that are phagocytosed by macrophages or neighboring cells, and the cellular contents do not diffuse into the surrounding environment and therefore do not cause an inflammatory response 17,18. Apoptosis can be triggered by endogenous and exogenous pathways. The initiators of the intrinsic pathway are BAK and BAX, which are regulated by pro-apoptotic proteins represented by BAD or anti-apoptotic proteins represented by BCL-2. BAK/BAX oligomers can form pores in the mitochondrial membrane that allow cytochrome C to be released into the cytoplasm. Cytochrome C binds to Apaf-1 and further recruits procaspase-9 to form apoptosome, causing apoptosis. On the other hand, membrane receptors, such as Tumor Necrosis Factor Receptor (TNFR), are the initiators of the extrinsic pathway and are induced by ligands to form apoptosis-inducing complexes that contain proteins (eg. Fas associated with death domain (FADD)) anchored on the cell membrane, which subsequently activate caspase-8, causing downstream cascades, leading to apoptosis, and ultimately lead to apoptosis 10. Apoptosis is involved in many pathological and physiological processes in the body and is one of the most frequently studied mechanisms in diabetes and diabetic complications. During different healing stages of diabetic wounds, apoptosis is abnormal in a variety of cells, including fibroblasts, KCs, ECs, EPCs, neutrophils, and macrophages. This results in uncontrollable wound inflammation, blocked angiogenesis, and impaired re-epithelialization. Therefore, apoptosis is a non-negligible orchestrator in remodeling the diabetic wound healing process.

### 2.1 Apoptosis of fibroblasts in diabetic wound healing

Fibroblasts are heterogeneous cells participating in the proliferation and remodeling of wound healing. Under normal circumstances, fibroblasts can synthesize and remodel a large amount of collagen extracellular matrix (ECM) and participate in the formation of granulation tissue, thereby repairing wound defects and providing necessary conditions for wound closure 19. In wound sites, fibroblasts can also transform into myofibroblasts, or regulate other adjacent cells by secreting a variety of cytokines to promote wound healing 20. Excessive apoptosis of fibroblasts leads to poor wound healing in diabetic hyperglycemic environments.

Non-coding RNAs (ncRNAs) are important molecules that do not encode proteins, but play biological functions such as gene expression regulation at the post-transcriptional level of RNA. At present, various ncRNAs represented by long non-coding RNAs (lncRNAs) and microRNAs (miRNAs) have also been reported to be involved in regulating the apoptosis of fibroblasts in diabetic wounds. Li et al. found that lncRNA H19 could recruit serum response factor (SRF) in the promoter region of connective tissue growth factor (CTGF) to increase the expression of CTGF, thereby inhibiting apoptosis and contributing to diabetic wound healing 21. Meanwhile, lncRNA H19 inhibited the apoptosis of fibroblasts by regulating miR-29b to increase fibrillin gene 1 (FBN1) expression and by regulating miR-152-3p to increase the phosphatase and tensin homolog (PTEN) expression 22,23. Zhang et al. reported that the expression of miR-27-3p was increased in fibroblasts under a high glucose environment, while the inhibition of miR-27-3p reduced the apoptosis of fibroblasts 24. Zhao et al. reported that miR-103 was increased in diabetic wounds and could inhibit the proliferation of fibroblasts and promote the apoptosis of fibroblasts, which is related to delayed diabetic wound healing 25. He et al. reported that lncRNA CASC2 was lower in diabetic wounds, and its overexpression could inhibit apoptosis and facilitate diabetic wound healing 26.

Besides, advanced glycation end products (AGEs) could induce oxidative stress and inflammatory responses in fibroblasts, which were closely related to fibroblast apoptosis 27. Lao et al. found that tissue inhibitor of metalloproteinases-1 (TIMP-1) levels were significantly reduced in AGEs-treated fibroblasts, and TIMP-1 could effectively reduce apoptosis 28. Notably, CTGF expression was inhibited in diabetic wounds, which may be related to high glucose-mediated fibroblast apoptosis. Overexpression of CTGF inhibited apoptosis by activating the mitogen-activated protein kinase (MAPK) signaling pathway in diabetic wounds. Also, growth differentiation factor 11 (GDF11) could regulate phosphorylated yes-associated protein (YAP) and phosphorylated Smad2/3, thereby promoting CTGF expression to improve diabetic wound healing 21,29.

### 2.2 Apoptosis of keratinocytes in diabetic wound healing

Re-epithelialization is an important step in the wound healing process. During re-epithelialization, KCs, the major cellular constituent of the epidermis, migrate to the top of the granulation tissue, and meet KCs from the other edge of the wound, thereby closing the wound 30. In diabetic wounds, excessive apoptosis of KCs is a key cellular event in wound closure.

Nuclear factor-E2-related factor 2 (Nrf2) is critical in protecting cells and maintaining cellular homeostasis when cells are under stress 31. The activation of Nrf2 and its downstream antioxidant genes could effectively reduce KCs apoptosis 32. Under a high glucose environment, Sun et al. reported that Nrf2 inhibition decreased its downstream heme oxygenase 1 (HO-1) expression, which was associated with decreased KCs proliferation, increased KCs apoptosis, and impaired diabetic wound healing 33. Furthermore, xanthohumol could reduce KCs apoptosis caused by high glucose, leading to accelerated diabetic wound healing. Specifically, xanthohumol increased the Nrf2 expression and promote its nuclear translocation in KCs, which was related to the activation of AMP-activated protein kinase α (AMPKα) and covalent modification of The kelch-like ECH-associated protein 1 (Keap1) 34. Guo et al. showed that a self-perpetuating feedback loop with Keap1/p-Nrf2 increased the oxidative stress-mediated apoptosis of ECs in diabetes 35. Oxidative stress, the dysfunction and apoptosis of KCs could be weakened by Astragaloside IV via activating the TGF-β/Smad signaling pathway 36.

Matrix metalloproteinase 9 (MMP9), a proteolytic enzyme belongs to the MMP family, is another important cause of KCs apoptosis. MMP9 could increase FasL expression and exert a pro-apoptotic effect on KCs through the FasL/Fas signaling pathway, which delayed diabetic wound healing 37. Zhou et al also found that berberine could down-regulate MMP9 in diabetic wounds, thereby accelerating diabetic wound healing 38. In addition, connexin31.1 (Cx31.1) is a gap junction protein and it was reported to be associated with KCs apoptosis in diabetic wounds 39. In a recent study, miR-204-3p was reported to improve KCs proliferation and reduce KCs apoptosis by targeting Krüppel-like factor 6 (KLF6) under a high glucose environment, thereby contributing to diabetic wound healing 40. Zinc finger transcription factor 148 (ZNF148) -activated metastasis associated in lung adenocarcinoma transcript 1 (MALAT1) could promote ZNF148 level by targeting miR-106a-3p, thus posing a positive feedback loop in modulating HaCaT cell proliferation, migration, and apoptosis 41.

### 2.3 Apoptosis of endothelial cells and endothelial progenitor cells in diabetic wound healing

Angiogenesis is a typical biological process in wound healing, involving key steps such as ECs activation and proliferation 42. Hyperglycemia can damage ECs and cause excessive apoptosis and dysfunction of ECs, which is one of the important pathological mechanisms in various diabetes complications 43. In diabetic wounds, excessive apoptosis of ECs can cause angiogenesis disorder, contributing to delayed wound healing.

Therefore, it is an intriguing issue to protect ECs from apoptosis in a high-glucose environment, consequently promoting angiogenesis and improving diabetic wound healing. Firstly, various organic compounds and extracts from traditional Chinese medicine are promising candidates. Vitamin D could both reduce the apoptosis rate and improve the vitality of ECs 44. The mechanism involved the reduction of endoplasmic reticulum stress. Huang et al. showed that ECs apoptosis induced by a high glucose environment could be alleviated by resveratrol 45. In this process, resveratrol acted as silencing information regulator 2 related enzyme 1 (SIRT1) agonist to stimulate the expression of c-Myc by promoting forkhead box transcription factor O1 (FOXO1) degradation, thereby protecting ECs. Correspondingly, ginsenoside Ginsenoside Rg1 (Rg1) also up-regulated SIRT1 expression and alleviated ECs apoptosis 46. In addition, ginsenoside Rg1 also relieved the inhibitory regulation of miR-23a on Interferon regulatory factor-1 (IRF-1), and then IRF-1 could increase the level of inducible NO synthase (iNOS) and promoted angiogenesis 47. Lei et al. reported that Panax notoginseng saponins promoted ECs proliferation, migration, and tubule formation, and inhibited ECs apoptosis, and ultimately improved diabetic wound healing 48. Umbilical cord mesenchymal stem cells (UCMSCs)-derived exosomal circHIPK3 protected HG-treated human umbilical vein endothelial cells (HUVECs) from cell apoptosis via miR-20b-5p/Nrf2/VEGFA axis 49.

Secondly, ncRNA is also an important target for promoting diabetic wound healing by reducing ECs apoptosis. In addition to miR-23a mentioned above, another study showed that miR-133b could cause the downregulation of epidermal growth factor receptor (EGFR), resulting in decreased ECs proliferation and increased apoptosis, which led to poor diabetic wound healing 50. Also, inhibition of miR-24-3p could reduce ECs apoptosis and promote angiogenesis by targeting phosphoinositide-3-kinase regulatory subunit 3 (PIK3R3) 51. Inhibition of miR-106a-5p could reduce ECs apoptosis and promote angiogenesis by targeting fibroblast growth factor 4 (FGF4) 52. Furthermore, lncRNA GAS5 and lncRNA KLF3-AS1 could accelerate diabetic wound healing by promoting ECs proliferation and tubule formation under a high glucose environment 53,54.

Notably, EPCs are precursor cells of ECs that can enter peripheral blood from the bone marrow and participate in the repair of damaged blood vessels, and have gradually attracted attention in diabetic wound healing 55. Increased apoptosis of EPCs in diabetes-induced hyperglycemia is associated with poor diabetic wound healing 56. Fan et al. reported that procyanidin B2 reduced the apoptosis of EPCs, and that the underlying mechanism might be related to the Nrf2 activation and the oxidative stress attenuation 57. Yang et al. found that exendin-4 increased EPCs viability and reduced apoptosis by inhibiting the p38 MAPK pathway, reducing endoplasmic reticulum stress and reactive oxygen species (ROS) 58. Zhang et al. applied platelet-rich plasma (PRP) in conjunction with the transplantation of EPCs to treat diabetic wounds. PRP could effectively reduce the apoptosis of EPCs with Notch1 signaling pathway activation phenotype 59. Furthermore, among ncRNAs, Gao et al. reported that miR-155 could increase apoptosis of EPCs by downregulating patched1 (PTCH1), and thus miR-155 may be a potential therapeutic target 60. The expression of stimulator of interferon genes (STING), a key protein in innate immunity, was increased markedly in diabetes, while using STING inhibitor reversed the damaged function of ECs and suppressed apoptosis by inhibiting IRF3/NF-κB pathway 61. Circ-Snhg 11 was reduced in EPCs under high glucose (HG) condition, while overexpression of circ-Snhg 11 could restrain EC injury by HG, including increased apoptosis and abnormal vascular differentiation 62.

### 2.4 Apoptosis of neutrophils and macrophages in diabetic wound healing

Neutrophils and macrophages are the two main types of cells involved in the inflammatory phase of the wound healing process. Large numbers of neutrophils are beneficial for infection control and wound healing after wound formation. As wound healing proceeds, neutrophil infiltration needs to be inhibited to allow inflammation to subside, otherwise a large number of proteases and ROS produced by neutrophils will interfere with subsequent wound healing 63,64. With the progress of wound healing, apoptosis of neutrophils can cause migration and phagocytosis of macrophages. Meanwhile, macrophages will transform from pro-inflammatory M1 macrophages to anti-inflammatory M2 macrophages, which secrete various growth factors to transition the wound from the inflammatory phase to the proliferative phase 65. However, in diabetic wounds, neutrophils and macrophages with pro-inflammatory phenotypes are susceptible to being overactivated, while their proper apoptosis and shift to an anti-inflammatory phenotype are impaired 66. It has been found that M1 macrophages with a pro-inflammatory phenotype make up 80% of the cells at the edge of chronic wounds and are able to secrete more pro-inflammatory cytokines when stimulated by inflammatory irritants 67,68. This permits the continued accumulation of chronic inflammation in the wound and reduces the proliferation and migration of keratinocytes, fibroblasts, and endothelial cells 69, thus leading to the result of delayed healing. In diabetic wounds, the researchers found that neutrophil apoptosis was reduced. Yang et al. found that insulin promoted the apoptosis of neutrophils in diabetic wounds, and then triggered the phenotype polarization of macrophages to promote diabetic wound healing 70. In addition, neutrophil apoptosis could be promoted through the FasL-Fas signaling pathway after administering a hybrid biomaterial in diabetic wounds, thereby transforming macrophages into M2 type with anti-inflammatory effects 71. Moreover, phagocytosis of apoptotic cells by macrophages is also affected. M1 macrophages were significantly weakened in phagocytosis due to the influence of AGEs in a high-glucose environment 72. On the one hand, this hindered the transformation of M1 to M2 macrophages, and on the other hand, it could also lead to a failure to remove apoptotic cells from the wound in a timely manner, and the increased apoptotic cells might delay the repair process 73. Exosomal MALAT1 derived from human KCs could enhance HG-injured macrophage functions and reduce apoptosis by suppressing miR-1914-3p to activate milk fat globule-EGF factor 8 (MFGE8), leading to a facilitation of wound healing 74. Taken together, it is evident that both neutrophils and macrophages are disturbed in diabetic wounds. Reduced apoptosis of neutrophils, and suppressed numbers and polarization of macrophages, may partially explain the poor healing of diabetic wounds.

## 3. Roles of autophagy in diabetic wound healing

Autophagy is another widely studied PCD modality, different from apoptosis 75. Autophagy is the transport of materials such as cytoplasmic proteins or organelles from the cell to the lysosome, forming autophagolysosomes to degrade and recycle the contents. Depending on the mode of delivery, autophagy can be divided into macroautophagy, microautophagy, and chaperone-mediated autophagy 76. Autophagy is regulated by a variety of signaling pathways, among which the classic autophagy regulatory pathway is that the unc-51-like kinases1/2 (ULK1/2) complex phosphorylates multiple downstream factors, induces phagophore nucleation, and then light chain 3- II (LC3-II), autophagy-related protein 9 (ATG9), ATG12-ATG5-ATG16L1 complex and the class III phosphatidylinositol 3-kinase (PtdIns3K) complex extend the phagosome to form an autophagosome, and LC3-II is removed from the outer membrane of the autophagosome. Finally, autophagosomes fuse with lysosomes to form autophagolysosomes 77. When autophagy is functioning properly, it can maintain cell survival by degrading some components in the cytoplasm into substances required for metabolism during cell starvation stress, or protect cells by degrading damaged or senescent organelles during normal cellular activities 78. Conversely, when autophagy functions are abnormal, insufficient or excessive levels of autophagy can be detrimental. Autophagy dysfunction is related to the occurrence and development of various diseases, including diabetes and diabetes-related complications 79. Here we summarize the relevant studies on autophagy in fibroblasts, KCs, ECs, EPCs, macrophages, and neutrophils involved in the healing process of diabetic wounds.

### 3.1 Autophagy of fibroblasts in diabetic wound healing

For fibroblasts during wound healing, autophagy has a dual regulatory role. A certain level of autophagy is important for fibroblast survival and maintenance of fibroblast function, while excessive autophagy is detrimental to fibroblasts. In the study by Yoon et al., they reported that activated autophagy effectively suppressed oxidative stress-induced apoptosis in skin fibroblasts 80. Activation of autophagy protected skin fibroblasts from various environmental pollutants such as benzo[a]pyrene and cadmium chloride 81. In contrast, in the study by Shi et al., they reported that activated autophagy inhibited fibroblast proliferation and migration 82. The autophagy function of fibroblast is dysregulated in diabetic wounds. There are different effects of autophagy dysfunction in fibroblasts on diabetic wounds. Zeng et al. used AGEs to pretreat HUVECs and then isolated small extracellular vesicles (EVs) secreted by the cells to study their effects on skin fibroblasts. They found that miR-106b-5p expression was up-regulated in these small EVs, resulting in decreased extracellular regulated kinase 1/2 (ERK1/2) expression in fibroblasts, which further led to activation of autophagy with reduced collagen synthesis and delayed wound healing 83. However, in the study of Martínez-Martínez et al. and Ben-Yehuda et al., they reported that a compound with the dual action of nitric oxide donor and phosphodiesterase-5 inhibitor, TOP-N53, could promote the expression of p62/SQSTM1 and activate autophagy in fibroblasts, and thus accordingly mediated the diabetic wound healing 84,85.

### 3.2 Autophagy of keratinocytes in diabetic wound healing

Autophagy is very important for KCs, which not only participates in the migration and differentiation process of KCs (especially the terminal differentiation process) to maintain the integrity of the epidermis, but also protects KCs from various extracellular or intracellular damage factors. Autophagy associated with endoplasmic reticulum stress plays an important role in the differentiation of KCs 86. Besides, Zhang et al. reported that Bcl-2 19-kDa interacting protein 3 (BNIP3)-mediated autophagy contributed to KCs migration during wound healing, and KCs migration was significantly attenuated when autophagy was inhibited 87. In addition, Yan et al. found that activated autophagy could increase the directed migratory speed of KCs, thereby promoting wound healing 88.

In diabetic wounds, high glucose environment and AGEs inhibit KCs autophagy, thereby interfering with KCs function and promoting KCs apoptosis, resulting in delayed wound closure. Regarding the mechanism of autophagy inhibition in KCs, Liang et al. deciphered that the m6A reader protein YTH Domain Containing 1 (YTHDC1) was inhibited in KCs in a high glucose environment, and YTHDC1 interacted with ELAVL1/HuR to regulate the expression of autophagy receptor SQSTM1. When the expression of SQSTM1 was decreased, autophagy of KCs was inhibited and wound healing was delayed 89. The study by Li et al. showed that the p38/MAPK signaling pathway in KCs was inhibited in a high glucose environment, which affected the autophagy function and led to an inhibition of cell migration. When the p38/MAPK signaling pathway was activated through MKK6 (Glu) overexpression, the migration of KCs could be promoted in an autophagy-dependent manner 90. TGF-β1 could phosphorylate SMAD2/SMAD3 in KCs, and promote autophagy by activating the SMAD signaling pathway, thereby improving diabetic wound healing 91. In addition, Feng et al. reported that the expression of STING was significantly up-regulated in KCs of diabetic wounds, which was related to autophagy dysfunction 92. They used rapamycin to induce autophagy, which effectively reduced the STING expression in KCs and contributed to wound healing.

### 3.3 Autophagy of endothelial cells and endothelial progenitor cells in diabetic wound healing

Autophagy of ECs and EPCs can enhance the apoptosis resistance of cells, thereby maintaining cell survival. Melatonin can promote autophagy and inhibit ECs apoptosis in an autophagy-dependent manner 93. Ma et al. reported that exogenous hydrogen sulfide ameliorated high glucose-induced EPCs dysfunction by promoting autophagy 94. In addition, activation of autophagy also plays an important role in the induction of angiogenesis. Notably, the angiogenesis capability could be strengthened by endothelial autophagy through some drugs represented by triazole derivatives 95.

In diabetic wounds, ncRNAs are important targets for regulating the autophagy function of EPCs under high glucose environment. The overexpressed circ-Klhl8 regulated the expression of its downstream miR-212-3p and silencing information regulator 2 related enzyme 5 (SIRT5), this pathway could activate autophagy in EPCs and improve cell survival, ultimately promoting diabetic wound healing 96. Besides, mmu_circ_0000250 could suppress miR-128-3p and upregulate SIRT1, thereby activating autophagy in EPCs, reducing apoptosis, and promoting diabetic wound healing 97. In addition, the autophagy function of EPCs could be impaired by AGEs in a high-glucose environment, which disturbed diabetic wound healing. Jin et al. found that melatonin could activate autophagy in EPCs, thereby reducing AGEs-induced apoptosis, promoting cell survival, and ultimately accelerating diabetic wound healing 98.

### 3.4 Autophagy of neutrophils and macrophages in diabetic wound healing

Autophagy can promote the increase and polarization of M2 macrophages and promote tissue repair 99. Several methods have been reported to promote autophagy in macrophages during wound healing. Xie et al. reported that stem cell exosomes were able to stimulate autophagy in macrophages to promote wound healing 100. Furthermore, Chiu et al. found that far-infrared could induce autophagy in macrophages, which led to the NACHT, LRR, and PYD domains-containing protein 3 (NLRP3) inflammasome inhibition and accelerated wound healing 101. In addition, autophagy is also actively involved in neutrophil-specific functions, including degranulation and formation of neutrophil extracellular traps (NETs) 102.

However, the regulatory role of autophagy on macrophages in diabetic wounds has been sporadically reported. Several studies have shown that there are different scenarios for the promotion or inhibition of autophagy on macrophages during diabetic wound healing. For example, Xie et al. found that the autophagy function of macrophages in diabetic wounds was impaired and weakened intracellular clearance of *S. aureus*, which significantly delayed diabetic wound healing 103. In contrast, Song et al. reported that negative pressure wound therapy could inhibit autophagy of macrophages with a reduced ratio of LC3-II/LC3-I and the expression of Beclin-1, and contributed to accelerated diabetic wound healing 104. Moreover, AGEs could activate autophagy and interfere with diabetic wound healing by stimulating the polarization of macrophages towards the M1 type 105. Therefore, autophagy seems to have a dual regulatory effect in macrophages under a high glucose environment, which may be related to the level of intracellular autophagy and needs to be further explored.

## 4. Roles of other types of programmed cell death in diabetic wound healing

In addition to apoptosis and autophagy, other emerging PCD types, such as pyroptosis, necroptosis, ferroptosis and cuproptosis, have been shown to be important biological processes involved in modulating various physiological and pathological conditions *in vivo*.

Pyroptosis is a new type of PCD characterized by cell swelling, pyroptosome formation, cell membrane rupture and the release of a large number of pro-inflammatory factors, which can cause a strong inflammatory response 106. Evidence has shown that pyroptosis in ECs is tightly connected with wound healing. In 2022, Zhang et al. reported pyroptosis inhibition in ECs by down-regulating the Cx43/ROS signaling pathway through bioactive glass, thereby promoting wound healing 107. In diabetic wounds, the activation of pyroptosis might be an important reason for the uncontrollable inflammatory response in wounds. Pastar et al. reported that pyroptosis and AIM2-inflammasome were activated by intracellular S. aureus due to Perforin-2 suppression, thereby prolonging diabetic wound healing 108. Besides, Exosomal lncRNA H19 derived from hair follicle mesenchymal stem cells (MSCs) enhanced HaCaT cellular bioactivity, and could efficiently accelerate diabetic healing *in vivo*, which was achieved by inhibiting NLRP3 pyroptosis 109. Apoptosis and pyroptosis of diabetic foot ulcer (DFU) fibroblasts could be restrained by overexpressing MALAT1 or knocking down miR-374a-5p 110.

Necroptosis is another unique type of PCD characterized by cell lysis, so necroptosis can cause an inflammatory response similar to that of pyroptosis. Necroptosis is usually activated when the apoptotic pathway is blocked 9. Necroptosis is abundant in chronic wounds and is denoted as an important factor in affecting wound healing 111. Yang et al. found that Silencing information regulator 2 related enzyme 3 (SIRT3) expression was reduced in diabetic wounds and prolonged wound healing, and the mechanism was associated with impaired mitochondrial function, increased oxidative stress, and increased necroptosis 112. In this study, the high expression pattern of necroptosis, with impaired mitochondrial function, increased oxidative stress, significantly prolonged diabetic wound healing process, in skin wounds of SIRT3-/- diabetic mice compared with WT phenotype.

Ferroptosis is a novel PCD that has been gradually recognized in recent years. Ferroptosis essence is the metabolic disorder of intracellular lipid oxides and the production of a large amount of ROS under the catalysis of iron ions, thereby causing cell death 113. There is a clear relationship between ferroptosis and inflammation, and this mediates the occurrence and development of various diseases 114. The levels of ROS, lipid peroxides, and ferroptosis-related proteins in fibroblasts and ECs in a high-glucose environment were significantly increased, which was associated with decreased cell survival and migration 115. Wei et al. confirmed that senile fibroblasts in diabetic wounds were rebellious to ferroptosis, which could be cured by overexpressing Nuclear Receptor Coactivator 4 (NCOA4) and thus led to quicker wound healing. Under ferroptosis inhibition, the PI3K/AKT signaling pathway was activated in diabetic wounds, and oxidative stress and inflammation were controlled, resulting in improved diabetic wound healing 116. Chen et al. demonstrated that HG-induced ferroptosis of HUVECs could be weakened by overexpressing circ-ITCH or co-cultured HUVECs with exosomal circ-ITCH from bone marrow mesenchymal stem cells (BMSCs), with the recruitment of TBP-associated factor 15 (TAF15) protein and the activation of the Nrf2 signaling pathway 117. After platelet-rich plasma intervention, ulcer wound tissue represented a decreased level of inflammatory cytokines and ferroptosis-related proteins/genes, and increased expression of CD31 and vascular endothelial growth factor (VEGF) 118.

In 2022, cuproptosis was discovered and attracted widespread attention. Especially, cuproptosis is emerging as a novel and striking molecular event in cellular biological processes. Similar to ferroptosis caused by the accumulation of iron, cytotoxicity could also occur when the copper concentration in the body exceeds the standard, causing cell death 119. To the best of our knowledge, the role of cuproptosis in diabetic wounds has not been reported. Further studies are needed to elucidate the possible role and mechanisms of cuproptosis in diabetic wounds and to provide novel perspectives for the treatment of diabetic wounds.

## 5. Discussion and prospect

Diabetes not only brings great troubles and challenges for patients and clinicians. Diabetes is prone to multiple complications, and diabetic wounds are one of the most common diabetic complications. The definition, precipitating factors, pathophysiology, clinical characteristics, treatment and prognosis comparison of normal wound healing with diabetic wound healing are presented in **Table 1**. Diabetic wounds have the characteristics of poor healing and secondary infection, which even threaten the life of patients in serious cases. It is important to understand the molecular mechanisms of diabetic wounds. We emphasize the current studies of PCD mechanisms for better diabetic wound healing outcomes.

There is an increased level of apoptosis in fibroblasts, KCs, ECs, and EPCs in diabetic wounds. Excessive apoptosis will affect epithelialisation, collagen deposition, and other important processes in diabetic wound healing, which will lead to delayed wound closure. Reduced levels of neutrophil apoptosis and inhibition of the conversion of M1 to M2 macrophages contribute to delayed healing of diabetic wounds. On the other hand, appropriate levels of autophagy can effectively prevent apoptosis and thus promote cell survival. However, the autophagy level of KCs, ECs, and EPCs is usually reduced in diabetic wounds. Notably, some studies have pointed to a possible dual regulatory role of autophagy in fibroblasts and macrophages. Furthermore, increased pyroptosis, necroptosis, and ferroptosis in diabetic wounds are also associated with uncontrollable inflammation, which in turn reduces cell survival and interferes with diabetic wound healing.

At present, there are still some perspectives worth pondering in this field. Firstly, PCD types, such as apoptosis, autophagy, pyroptosis, necroptosis, and ferroptosis, are commonly found in diabetic wounds. However, current studies have reported more on the role of apoptosis and autophagy in diabetic wounds, but less on other types of PCD. this may be due to the fact that apoptosis and autophagy were identified earlier and studied more intensively than other types of PCD. Meanwhile, it is also possible that apoptosis and autophagy indeed play a more central role in diabetic wound healing than other types of PCD. Therefore, more relevant studies are needed to clarify the role of the more newly discovered PCD types in diabetic wounds. Secondly, as mentioned above, it is still unclear whether there is a dominant PCD type in diabetic wound healing, and how different PCD types promote or inhibit each other in diabetic wound healing. The various types of PCD do not exist independently and may influence cell fate *in vivo* by regulating each other. In the future, the interrelationship and dominance of various PCDs in diabetic wound healing need to be determined more to clarify the mechanisms of multi-cellular and multi-factor regulation of death forms in diabetic wounds.

Thirdly, current related research mainly focuses on the selection of treatment methods for diabetic wounds targeting PCD, but specific molecular mechanism research is needed to better clarify the relationship between PCD and the pathological mechanism of diabetic wounds. Also, it is a good approach to search for PCD-related genes with marker properties at different stages and in different cells during the progression of diabetic wounds. Lastly, there is a lack of systematic studies on whether the types and roles of PCD differ in different parts of the body during diabetic wound healing, and how the types and roles of PCD change at different stages of healing. Furthermore, elucidation of the relationship between cell-cell communication and PCD and the relationship between epigenetics and PCD will contribute to a better understanding of the mechanism of PCD in diabetic wound healing. For PCD-targeted drugs, current research has focused on modulating one specific PCD type, so exploring drugs that can target and modulate multiple PCD types simultaneously, or combining different drugs that target PCD types, may be a new direction in the search for therapeutic approaches to improve diabetic wound healing.

On the other hand, as far as we know, almost all current research on PCD in diabetic wound healing are remaining at the level of basic research, and no drugs targeting PCD have successfully accomplished translation to the clinic. We consider that this may be explained by the following reasons: First, there is a complex PCD phenomenon among various cells in diabetic wounds. Apoptosis and autophagy may play a dual regulatory role in diabetic wounds, which increases the difficulty of applying drugs regulated these two kinds of PCD in clinical practice. Meanwhile, more studies are still needed about the situation of several other PCDs in diabetic wounds and the related therapeutic methods. Secondly, since PCD is extensively involved in regulating a wide range of biological processes, when selecting drugs, consideration should be given not only to their effectiveness for wound healing, but also to their safety. Thirdly, the major diabetic animal models used at present include STZ-induced diabetic animal models and transgenic animal models, but their wound models do not exactly reflect the clinical wound healing process. Moreover, considering that animal wounds are easily interfered with by external environments and infections, they may not be able to accurately reflect the actual effects of drugs on wound healing, which also affects the PCD-based therapeutic assessment. Lastly, more rigorous clinical studies are needed to evaluate the effectiveness and safety of PCD-based therapy in diabetic wound healing.

## 6. Conclusion

In conclusion, this article focuses on the role of various types of PCD in diabetic wound healing and provides a summary to better elucidate the molecular mechanisms of diabetic wound healing. In diabetic wounds, PCD including apoptosis, autophagy, pyroptosis, necroptosis and ferroptosis can change the activity and function of immune cells (including neutrophils and macrophages) and skin constituent cells (including fibroblasts, KCs, ECs, and EPCs). As a result, the inflammatory response, epithelialisation, granulation, and other pathophysiological processes in diabetic wounds are affected, which eventually leads to delayed wound closure. In the near future, more elucidation of PCD and diabetic wounds will help uncover new approaches to more effective treatment of diabetic wounds.

## 7. Abbreviation

PCD: Programmed Cell Death; KCs: Keratinocytes; ECs: Endothelial Cells; EPCs: Endothelial Progenitor Cells; AIM2: Absent in Melanoma 2; GSH: Glutathione; GPX4: Glutathione Peroxidase 4; TCA: Tricarboxylic Acid; TNFR: Tumor Necrosis Factor Receptor; FADD: Fas associated with death domain; ECM: extracellular matrix; ncRNAs: Non-coding RNAs; lncRNAs: Long non-coding RNAs; miRNAs: MicroRNAs; SRF: Serum response factor; CTGF: Connective tissue growth factor; FBN1: fibrillin gene 1; PTEN: Phosphatase and tensin homolog; AGEs: advanced glycation end products; TIMP-1: tissue inhibitor of metalloproteinases-1; MAPK: Mitogen-activated protein kinase; GDF11: growth differentiation factor 11; YAP: Yes-associated protein; Nrf2: Nuclear Factor Erythroid2-Related Factor 2; HO-1: Heme Oxygenase 1; AMPKα: AMP-activated protein kinase α; KEAP1: The Kelch-like ECH-associated protein 1; MMP9: The matrix metalloproteinase 9; Cx31.1: Connexin31.1; KLF6: Krüppel-like factor 6; ZNF148: zinc finger transcription factor 148; MALAT1: Metastasis associated in lung adenocarcinoma transcript 1; HG: high glucose; SIRT1: Silencing information regulator 2 related enzyme 1; FOXO1: Forkhead box transcription factor O1; Rg1: Ginsenoside Rg1; IRF-1: Interferon regulatory factor-1; iNOS: inducible NO synthase; UCMSC: umbilical cord mesenchymal stem cells; HUVECs: human umbilical vein endothelial cells; EGFR: epidermal growth factor receptor; PIK3R3: Phosphoinositide-3-kinase regulatory subunit 3; FGF4: fibroblast growth factor 4; ROS: Reactive Oxygen Species; PRP: platelet-rich plasma; PTCH1: Patched1; STING: stimulator of interferon genes; MFGE8: milk fat globule-EGF factor 8; ULK: Unc-51-like kinases; LC3-II: light chain 3- II; ATG9: autophagy-related protein 9; PtdIns3K: phosphatidylinositol 3-kinase; EV: extracellular vesicles; ERK: extracellular regulated kinase; BNIP3: Bcl-2 19-kDa interacting protein 3; YTHDC1: YTH Domain Containing 1; SIRT5: Silencing information regulator 2 related enzyme 5; NLRP3: The NACHT, LRR, and PYD domains-containing protein 3; NETs: Neutrophils produce neutrophil extracellular traps; MSCs: mesenchymal stem cells; DFU: diabetic foot ulcer; SIRT3: Silencing information regulator 2 related enzyme 3; NCOA4: Nuclear Receptor Coactivator 4; BMSCs: bone marrow mesenchymal stem cells; TAF15: TBP-associated factor 15; VEGF: vascular endothelial growth factor.

## Figures and Tables

**Figure 1:**
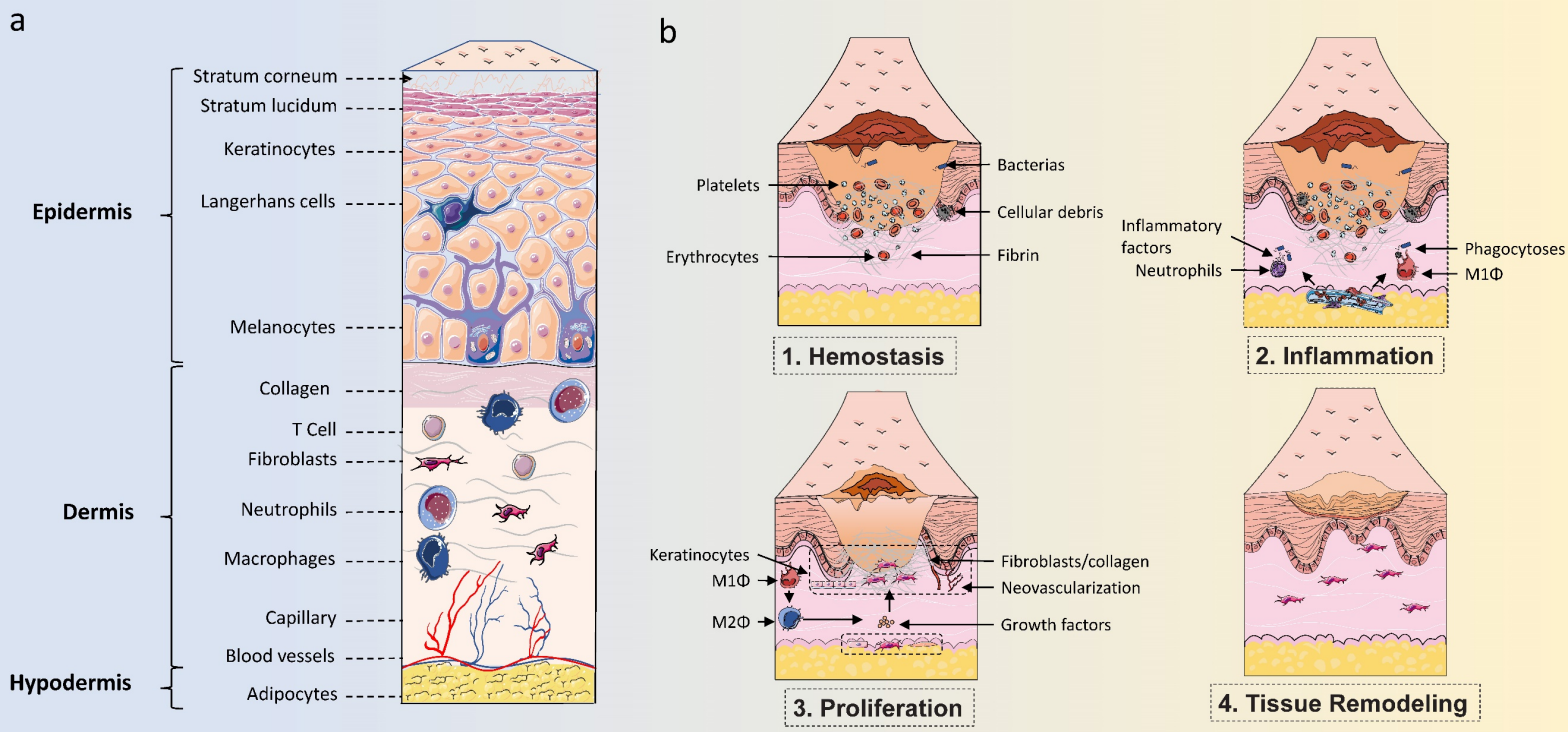
**Skin structure and healing process. (A)** The structure of the skin consists of three parts: the epidermis, the dermis, and the hypodermis. The epidermis is an important protective layer of skin, mainly consisting of keratinocytes and non-keratinocytes. Non-keratinocytes mainly include Langerhans cells and melanocytes, while keratinocytes are the main cells that make up the structure of each layer. The dermis, which consists of dense connective tissue, lies beneath the epidermis and is mainly composed of fibroblasts, collagen, and various immune cells. The dermis is the primary site within the skin where immune responses occur and where skin density and firmness are maintained. The hypodermis consists of loose connective tissue and fat cells that lie beneath the dermis, maintaining body temperature and cushioning pressure.** (B)** The process of wound healing can be divided into four periods: hemostasis, inflammation, proliferation, and tissue remodeling. Multiple cell types, including endothelial cells, keratinocytes, fibroblasts, and various immune cells, work together to coordinate the skin wound healing process.

**Figure 2:**
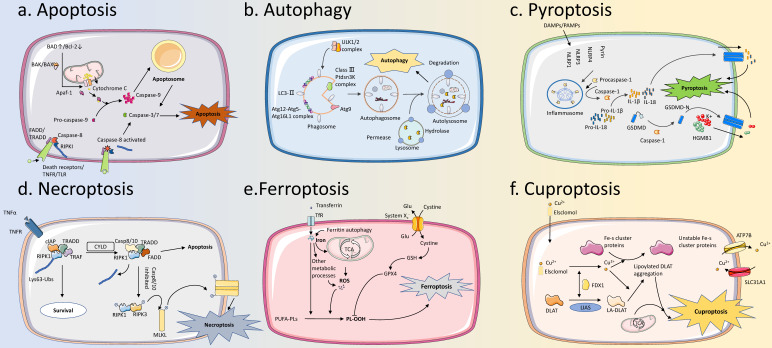
** Brief description of the PCD process. (A) Apoptosis:** The pathways of apoptosis can be divided into intrinsic and extrinsic pathways. These two pathways are regulated by BAD/BCL-2 and signaling through membrane receptors (for example Death Receptors/TNFR/TLR), respectively, and then intersect at Caspase 3/7, leading to apoptosis. **(B) Autophagy:** The ULK1/2 complex is activated to phosphorylate multiple downstream factors, induces the formation of the phagosome, and further envelopes cargo to form the autophagosome. Autophagosome binds to the lysosome, the internal cargos are degraded by hydrolytic enzymes within the lysosome, and nutrients enter recirculation. **(C) Pyroptosis:** DAMPs/PAMPs stimulate intracellular receptors to form Inflammasome, which then activates Caspase-1. Activated Caspase-1 can cleave Pro-IL-1β, Pro-IL-18, and GSDMD to form active forms, and activated GSDMD-N forms oligomers to form pores in the membrane. IL-1β, IL-18, and HGMB-1 can be released from the pores, leading to pyroptosis. **(D) Necroptosis:** Activation of TNFR and other receptors induces the formation of the cIAP-TRADD-RIPKI-TRAF complex. Upon detection of the death signal, CYLD deubiquitinates RIPKI and induces the formation of the Caspase-8/10-TRADD-RIPKI-TRAF complex. Caspase-8/10 can be activated to induce apoptosis. When Caspase-8/10 is inhibited, the complex can trigger a downstream phosphorylation cascade reaction that leads to necroptosis. **(E) Ferroptosis:** Iron accumulation and ROS production from multiple metabolic pathways can lead to intracellular lipid peroxidation, which in turn triggers ferroptosis. System Xc- can inhibit ferroptosis by increasing the transport of cystine. **(F) Cuproptosis:** Copper ion carriers, such as Elsclomol, transport copper into the cells. Copper can bind to lipoylated mitochondrial enzymes in the TCA cycle then lead to their aggregation, and can also lead to destabilization of Fe-S clusters, which in turn leads to cell death. PCD, Programmed Cell Death; DAMPs, Damage-Associated Molecular Patterns; PAMPs, Pathogen-Associated Molecular Patterns; ROS, Reactive Oxygen Species.

**Figure 3:**
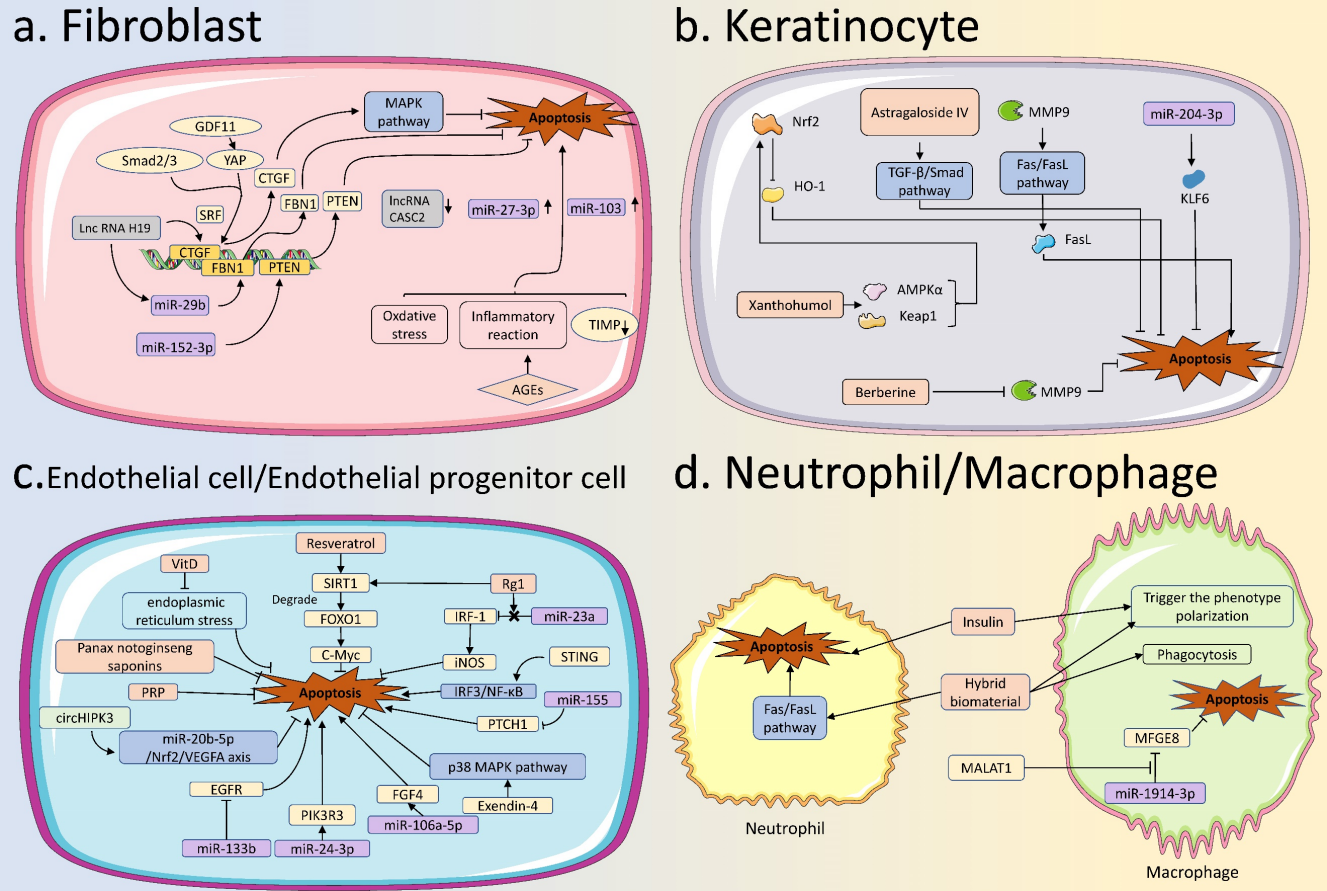
** Apoptosis in various skin cells in diabetic wound healing. (A) Apoptosis in fibroblasts:** GDF11, miR-29b, miR-152-3p, and lncRNA H19 are involved in apoptotic regulation of fibroblasts in diabetic wound healing by regulating the expression of targeted genes. MiR-103, miR-27-3p, and lncRNA CASC2 are also aberrantly expressed in fibroblasts. AGEs can regulate apoptosis by increasing intracellular levels of oxidative stress, and inflammatory reaction, and decreasing levels of TIMP. **(B) Apoptosis in KCs:** Various drugs, such as Astragaloside IV, Xanthohumol, and Berberine, can inhibit the apoptosis of keratinocytes in diabetic wounds. Some specific miRNAs, such as miR-204-3p, can inhibit the apoptosis of KCs to promote the healing of diabetic wounds. **(C) Apoptosis in ECs:** Some factors, represented by Resveratrol, VitD, STING, miR-133b, miR-106a-5p, miR-155, and circHIPK3, can modify the healing process of diabetic wounds by regulating apoptosis of ECs. **(D) Apoptosis in neutrophils and macrophages:** Normal apoptosis of immune cells is essential for the normal healing of diabetic wounds. In diabetic wounds, insulin promotes the apoptosis of neutrophils and also affects the phenotypic transformation of macrophages. The hybrid biomaterial can facilitate apoptosis of neutrophils and also influence phagocytosis and phenotypic transformation of macrophages. The exosomal MALAT1 can enhance diabetic wound healing by inhibiting macrophage apoptosis. AGEs, Advanced Glycation End products; TIMP, Tissue Inhibitor of Metalloproteinase; KCs, keratinocytes; Ecs, Endothelial Cells.

**Figure 4:**
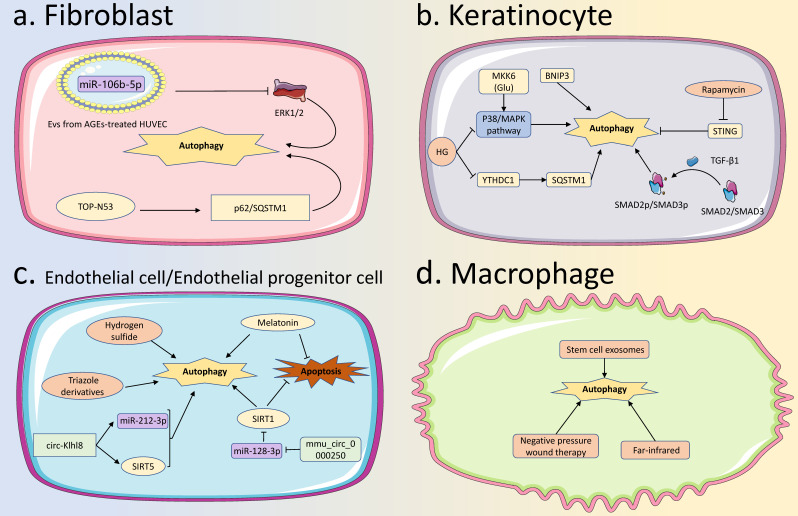
**Autophagy in various skin cells in diabetic wound healing. (A) Autophagy in fibroblasts:** High expression of miR-106b-5p in EVs from AGEs-treated HUVEC, reduces the ERK1/2 expression to activate autophagy in fibroblasts, leading to delayed diabetic wound healing. TOP-N53 can promote fibroblast p62/SQSTM1 expression to activate autophagy and improve diabetic wound healing. **(B) Autophagy in KCs:** High glucose can affect the P38/MAPK pathway and YTHDC1 to inhibit KCs autophagy, causing delayed diabetic wound healing, while BNIP3, TGF-β1, and rapamycin can activate autophagy to accelerate diabetic wound healing. **(C) Autophagy in ECs:** Several factors have been found to promote the healing of diabetic wounds by activating autophagy in ECs. Including triazole derivatives, hydrogen sulfide, circ-Klhl8, and mmu_circ_0000250. SIRT1 and melatonin can activate the autophagy of ECs and inhibit their apoptosis, thus inducing the healing of diabetic wounds. **(D) Autophagy in macrophages:** Stem cell exosomes, far-infrared, and negative pressure wound therapy, can all activate autophagy in macrophages to promote the healing of diabetic wounds. EVs, Extracellular Vehicles; AGEs, Advanced Glycation End products; HUVECs, Human Umbilical Vein Endothelial Cells; KCs, Keratinocytes; ECs, Endothelial Cells.

**Table 1:** The definition, precipitating factors, pathophysiology, clinical characteristics, treatment and prognosis comparison between normal wound healing and diabetic wound healing.

	Normal wound healing	Diabetic wound healing
**Definition**	A dynamic repair process that occurs after a breach of anatomical integrity	Wound healing occurring on the basis of diabetes
**Precipitating factors**	Various external physical and chemical injuries	External injurious factors and the presence of internal hyperglycemia
**Pathophysiology**		
**Hemostasis processess**	Platelet aggregation Release of vasoactive substances	Platelet hyperaggregation Accelerated local vascular lesions
**Inflammation processess**	Moving and concentrating immune cells toward the wound Release of protease and various cytokines	Unbalanced ratio of M1 and M2 macrophages Uncontrolled inflammatory response
**Proliferation processess**	Epithelialisation Granulation	Decreased angiogenesis Decreased collagen deposition
**Remodeling processess**	Collagen reorganization Scar tissue remodeling	Interrupted processes of granular tissue to scar tissue Decreased tensile strength
**Clinical characteristics**	Timely wound closure Generally healing without any issue	Delayed wound closure Susceptibility to infection
**Treatment**	Fundamental treatment Vacuum sealing drainage Wound dressings Autologous skin graft Synthetic replacement for tissues	Symptomatic treatment Hyperbaric oxygen therapy Silver nanoparticles Glucose control Infection prevention
**Prognosis**	Favorable prognosis Structural and functional recovery	Unfavourable prognosis Life-threatening in some cases
